# A Review of the Role of Social Cognition in Major Depressive Disorder

**DOI:** 10.3389/fpsyt.2014.00179

**Published:** 2014-12-11

**Authors:** Michael James Weightman, Tracy Michele Air, Bernhard Theodor Baune

**Affiliations:** ^1^Discipline of Psychiatry, School of Medicine, The University of Adelaide, Adelaide, SA, Australia

**Keywords:** social cognition, major depressive disorder, depression, facial affect, theory of mind

## Abstract

**Background:** Social cognition – the ability to identify, perceive, and interpret socially relevant information – is an important skill that plays a significant role in successful interpersonal functioning. Social cognitive performance is recognized to be impaired in several psychiatric conditions, but the relationship with major depressive disorder is less well understood. The aim of this review is to characterize the current understanding of: (i) the different domains of social cognition and a possible relationship with major depressive disorder, (ii) the clinical presentation of social cognition in acute and remitted depressive states, and (iii) the effect of severity of depression on social cognitive performance.

**Methods:** Electronic databases were searched to identify clinical studies investigating social cognition in a major depressive disorder population, yielding 31 studies for this review.

**Results:** Patients with major depressive disorder appear to interpret social cognitive stimuli differently to healthy controls: depressed individuals may interpret emotion through a mood-congruent bias and have difficulty with cognitive theory of mind tasks requiring interpretation of complex mental states. Social cognitive performance appears to be inversely associated with severity of depression, whilst the bias toward negative emotions persists even in remission. Some deficits may normalize following effective pharmacotherapy.

**Conclusions:** The difficulties with social interaction observed in major depressive disorder may, at least in part, be due to an altered ability to correctly interpret emotional stimuli and mental states. These features seem to persist even in remission, although some may respond to intervention. Further research is required in this area to better understand the functional impact of these findings and the way in which targeted therapy could aid depressed individuals with social interactions.

## Background

Correctly interpreting social information is a crucial part of successful interpersonal interaction. This requires synthesis of a broad range of verbal and non-verbal cues, including facial expressions, prosody in speech, body language, and the mental states of others (theory of mind). Together, these skills are referred to as social cognition and are an important component of cognitive functioning. Social cognition encompasses the identification, perception, and interpretation of socially important information (1), whilst the domain of theory of mind specifically refers to the ability to infer information regarding the thoughts, intentions, and feelings of others (2). This is further conceptualized into two distinct systems – cognitive theory of mind relates to interpretation of beliefs and intentions, whilst affective theory of mind consists of inferences regarding the emotional states of others (3).

Social cognitive impairment is widely recognized to be a key feature of several psychiatric diseases, such as schizophrenia (1) and autism (4). The impact of depression on social cognitive functioning is less well understood, although there is some evidence to suggest that a similar, albeit less severe, impairment of social cognition may be seen in patients with major depressive disorder. Whilst major depressive disorder is primarily characterized by emotional symptoms such as low mood and anhedonia (5), individuals with depression have also been found to display profound and pervasive impairments in interpersonal functioning (6). Nevertheless, depressed patients appear to be less severely impaired in social cognition than patients with schizophrenia or autism (7–9).

The majority of data from previous reviews investigating the relationship between depression and social cognition relate primarily to the emotional domain, particularly through facial expressions or affective theory of mind. One meta-analysis of eight studies found major depressive disorder to be significantly associated with impaired recognition of emotional facial expressions with a moderate overall effect size (10). A larger review indicated a reasonably consistent pattern of a negative interpretive bias of facial affect (11). It appears that depressed patients may not exhibit diminished recognition accuracy when interpreting facial expressions, but rather demonstrate an increased sensitivity toward sad expressions in comparison to happy ones. Other reviews have indicated that such abnormalities appear to respond to anti-depressant therapy and may actually be detectable prior to improvements in mood (12, 13).

To our knowledge, there is a lack of similar comprehensive reviews of behavioral studies in the domains of prosody, body language, and theory of mind to determine if these findings are consistent across all components of social cognition in major depressive disorder. Evidence from individual studies indicates that patients with major depressive disorder may also struggle with verbal cues, with two studies reporting a negative bias when depressed patients interpret prosodic stimuli (14, 15). Several authors who have tested theory of mind performance in depressed cohorts have found them to be impaired, albeit less severely than psychotic or autistic participants (7, 8). It is therefore reasonable to anticipate that the impairments in social cognition in major depressive disorder may translate across the different domains.

In addition to the available behavioral data, there is also a significant body of literature that exists on functional neuroimaging in patients with depression undergoing social cognitive testing. Much of this literature indicates a neural basis for the observed phenomenology, primarily centering around dysregulation of both the amygdala and ventromedial frontal cortex (16–19). In light of such functional changes, detailed examination is required to determine the extent to which altered processing of social stimuli may impact the quality of life or interpersonal success of an individual with major depressive disorder.

Better understanding of the nature of social cognition in major depressive disorder is important, as it will help to further characterize the phenotype of the disease and may offer explanation for the poor social functioning observed in depression. It is also important to consider the potential therapeutic implications, as previous reviews have suggested that impairments in social cognition may be reversible (12, 13). Improvement in social cognitive functioning may become a targeted component of the treatment for depression or a useful method of monitoring improvement.

The present review of the existing literature on social cognition and major depressive disorder aims to increase the understanding of: (i) the impact of depressed mood across different domains of social cognition including facial affect, prosody, body language, and theory of mind, (ii) the clinical presentation of social cognition in acute and remitted depressive states, and (iii) the effect of severity of depression on social cognitive performance.

## Methods

### Search strategy

An electronic search strategy was employed to identify published studies investigating the behavioral relationship between social cognition and depression. Pertinent original research articles were identified and retrieved via the Medline, Embase, and PsycINFO databases, as well as the reference lists of included articles. The search was limited to only the most recent English literature, taking into account the 10-year period from June 2003 to June 2013. The subject headings used across the different databases were “social cognition,” “social perception,” “depression,” “major depression,” “mood disorders,” “depressive disorder,” and “affective disorders.” This was combined with the key word search terms of “social cognition,” “social perception,” “social interaction,” “depression,” “mood disorder,” “affective disorder,” “melancholia,” and “dysthymia.” Truncated search terms were included where possible to maximize results, while inclusion of broad key words such as “mood disorder,” “melancholia,” or “dysthymia” was intended to enhance the sensitivity of the search.

### Study selection

**Figure 1:**
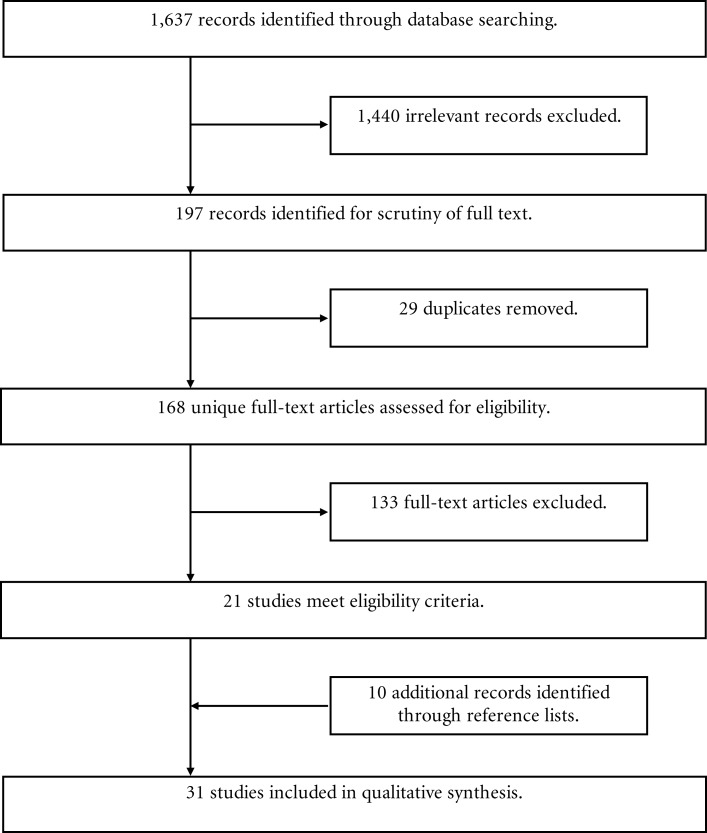
**Flowchart of article inclusion procedure during review of the literature**. From 1,637 individual records initially identified through the literature search of the Medline, PsychINFO, and Embase databases, 31 articles were included in the final analysis.

The titles and abstracts of the results generated from the three databases were screened to exclude irrelevant articles. The full articles of the remaining studies were downloaded and the methods inspected in detail to determine eligibility (Figure 1). The identified studies were included in the analysis if they met the following criteria: firstly, the diagnosis of an acute or remitted major depressive disorder was formally made according to a standardized interview; secondly, the patient population needed to be an adult study group, defined as participants aged 18 and above; thirdly, each study was required to report behavioral data relating to a social cognitive task in one or more of the following domains only – facial affect recognition, theory of mind, prosody, or body language interpretation.

Studies were excluded if the patient group was co-morbid with a separate psychiatric disorder, such as schizophrenia, dementia, a learning disorder, eating disorder, or pervasive developmental disorder. Studies were also excluded if the patient group demonstrated bipolar features. Functional MRI studies with no reported behavioral results were excluded. Once selected, the article’s reference list was then scoured for further relevant articles missed in the initial database searches.

## Results

Over the past decade, much has been published to further characterize the relationship between social cognition and major depressive disorder. Following a thorough search of the literature, a total of 31 articles were included in this review (Figure 1).

Social cognition is a broad term encompassing a diverse array of skills. The two most studied social cognitive domains are facial affect recognition and theory of mind, with the majority of included studies in this review investigating ability in these two areas. Some authors have applied a bimodal approach to assessing social cognition, using both a perceptual assessment of observable social information for facial affect with a more conceptual approach involving higher order functions for theory of mind (2, 7, 8, 20, 21). Others have included empathy (2, 22) among the domains of social cognition. No studies were uncovered using the above methodology that specifically investigated prosody of voice or body language, although body language was indirectly assessed in the tasks used by some authors (2, 22). This suggests a need for further research into the impact on other social cognitive domains in major depressive disorder.

### Major depressive disorder and function on specific domains of social cognition

The literature remains equivocal regarding overall differences in social cognitive performance between depressed and healthy cohorts. While there is some evidence to suggest that subjects with major depressive disorder may demonstrate poorer overall social cognitive performance compared to healthy controls (Table 1A), numerous other authors found no group difference on a gross measure of social cognition (Table 1B). This is further discussed below according to the domains of facial affect recognition, affective theory of mind, and cognitive theory of mind.

**Table 1:** **Case-control studies investigating differences in social cognitive performance between patients with major depressive disorder and controls**.

References	Aim	Method	*N* [MA ± SD; M:F]	Social cognition task	Mood diagnosis	Results
**(A) STUDIES INDICATING SIGNIFICANT DIFFERENCES BETWEEN ACUTELY DEPRESSED INDIVIDUALS AND HEALTHY CONTROLS**						
Anderson et al. (23)	To compare accuracy, discrimination, and bias in face recognition in current and remitted depression	Case-control GP sample	MDE = 30, rMDD = 99, HC = 101 [33.1 ± 10.5; 71M:159F]	FERT	DSM-IV	Significant difference between MDE, rMDD, and HC groups on facial affect recognition accuracy (*F*_2,225_ = 5.340, *P* = 0.005)
Cao et al. (21)	To investigate social cognitive performance in esophageal cancer patients with depression	Case-control	pMDD = 32, npMDD = 33, HC = 62 *n* = 127 [33.1 ± 10.5; 71M:159F]	RMET-R, FPT	BDI-II	Compared to HC, both MDD groups were impaired on affective ToM (*t* = 7.39, *P* < 0.01) and faux pas task (*t* = 13.75, *P* < 0.01)
		Esophageal cancer patient sample	
Csukly et al. (24)	To determine if depressed patients perceive emotion differently to controls and if this is due to emotional intensity and arousal	Case-control Clinical sample	MDD = 23, HC = 23 [48.4 ± 12.5; 18M:28F]	VHI	DSM-IV, ICD-10	MDD impaired at recognizing facial expressions compared to HC (*F*_46_ = 6.76, *P* = 0.02), particularly at low intensity or arousal
Csukly et al. (25)	To identify associations between depressive severity, maladaptive schemas, and facial affect recognition	Case-control Inpatient sample	MDD = 107, HC = 23 [41.1 ± 11.3; 16M:90F]	VHI	DSM-IV	MDD impaired at recognizing facial expressions compared to HC (*t* = 5.2, *P* < 0.0001)
Donges et al. (26)	To examine emotional awareness in depressed inpatients following a psychotherapy program	Prospective longitudinal case-control Inpatient sample	MDD = 22, HC = 22 [32.1 ± 8.6; 14M:30F]	LEAS	DSM-IV	MDD performed inferiorly to HC at emotional awareness of others (*F*_1,42_ = 5.5, *P* < 0.05)
						Performance of the MDD group improved over the 7 weeks of treatment (*F*_1,42_ = 5.6, *P* < 0.05)
Harkness et al. (27)	To determine if maternal history of depression impacts affective ToM performance	Case-control Outpatient sample	MDD = 61, HC = 30 [45.3 ± 14.5; 0M:91F]	RMET-R	DSM-IV	MDD performed significantly worse on affective ToM task than HC (*F*_1,91_ = 6.73, *P* = 0.01)
Langenecker et al. (28)	To evaluate emotion perception deficits in depressed women Outpatient sample	Case-control	MDD = 21, HC = 20 [30.9 ± 9.2; 0M:41F]	FEPT	BDI-II, HRSD	MDD performed inferiorly to HC in facial affect recognition accuracy (*F*_1,38_ = 6.40, *P* = 0.02)
Lee et al. (29)	To examine depressed patients’ abilities to identify mental states from affective eye expressions	Case-control Outpatient sample	MDD = 67, HC = 34 [42.7 ± 14.1; 0M:82F]	RMET-R	DSM-IV	Severe MDD less accurate than HC on affective ToM task (*t*_65_ = 2.24, *P* = 0.03); no difference between mild/moderate MDD and HC or MDD groups
Leppänen et al. (30)	To determine if depression biases the recognition of emotionally neutral faces	Case-control Inpatient sample	MDD = 18, HC = 18 [44.9 ± 9.9; 14M:22F]	PFA	ICD-10	MDD worse than HC in facial affect recognition accuracy (*F*_1,34_ = 9.1, *P* < 0.006), due to misidentification of neutral faces (*t*_34_ = 4.8, *P* < 0.001)
Surguladze et al. (31)	To investigate the accuracy and response bias of depressed people to affective facial expressions	Case-control Clinical sample	MDD = 27, HC = 29 [45.0 ± 11.6; 24M:32F]	FEEST	DSM-IV	MDD worse than HC in facial affect recognition accuracy (*F*_1,42_ = 26.2, *P* < 0.01)
Szily and Kéri (32)	To determine the impact of psychosis risk in depression on social cognition	Case-control Clinical sample	prMDD = 26, MDD = 42, HC = 50 [21.2 ± 7.3; 44M:73F]	RMET-R	DSM-IV	prMDD and MDD were less accurate than HC on RMET-R (*F*_2,230_ = 10.30, *P* < 0.001)
Wang et al. (8)	To determine if psychotic features in depression impact social cognitive performance	Case-control Inpatient sample	pMDD = 23, npMDD = 33, HC = 53 [26.8 ± 4.4; 47M:62F]	RMET-R, FPT	ICD-10	On RMET-R, pMDD inferior to both npMDD (*P* = 0.018) and HC (*P* = 0.000), npMDD superior to HC (*P* = 0.000)
						On FPT, HC superior to both pMDD (*U* = 0.000, *P* = 0.000) and npMDD (*U* = 128.500, *P* = 0.000), pMDD inferior to npMDD (*U* = 149.500, *P* = 0.000)
Wolkenstein et al. (2)	To investigate difference in social cognitive performance between depressed patients and controls	Case-control Outpatient sample	MDD = 24, HC = 20 [36.4 ± 10.8; 19M:25F]	RMET-R, MASC	DSM-IV	MDD inferior to HC in performance on MASC (*F*_1,42_ = 4.57, *P* < 0.05), but equal on RMET-R (*F*_1,42_ = 0.74, *P* < 0.40)
Zobel et al. (33)	To compare social cognitive performance between depressed patients and controls	Case-control Clinical sample	MDD = 30, HC = 30 [46.5 ± 12.0; 27M:33F]	BCPS	DSM-IV	MDD inferior to HC in BCPS sequence (*U* = 242.5, *P* = 0.001), FOQ (*U* = 294.0, *P* = 0.004), SOQ (*U* = 183.5, *P* = 0.001); and WE.EL sequence (*U* = 282.0, *P* = 0.012), FOQ (*U* = 297.0, *P* = 0.016), SOQ (*U* = 234.0, P = 0.001)
				WE.EL	
**(B) STUDIES INDICATING NON-SIGNIFICANT DIFFERENCES BETWEEN ACUTELY DEPRESSED INDIVIDUALS AND HEALTHY CONTROLS**						
Bazin et al. (7)	To evaluate a new social cognitive task in a clinical sample	Case-control Inpatient sample	MDD = 12, HC = 15 [36.6 ± 12.8; 36M:21F]	V-SIR, ToM comic	DSM-IV	MDD performed non-significantly worse on V-SIR compared to HC. No group effect for ToM comic test
Bediou et al. (34)	To compare how depressed and schizophrenic patients recognize facial affect	Case-control Clinical sample	MDD = 20, HC = 20 [32.9 ± 9.8; 42M:27F]	Self-created	DSM-IV	MDD and HC performed equally well on facial affect recognition
Bertoux et al. (20)	To evaluate a new social cognitive task to distinguish depression from frontotemporal dementia	Case-control Inpatient sample	MDD = 19, HC = 30 [65.1 ± 9.0; 47M:39F]	Mini-SEA (PFA, sFPT)	DSM-IV	On total mini-SEA and component scores, MDD performed equally to HC
Gollan et al. (35)	To identify differences in affective information processing between depressed patients and controls	Case-control Clinical sample	MDD = 37, HC = 29 [35.1 ± 9.3; 34M:32F]	PFA	DSM-IV	MDD and HC performed equally in facial affect recognition and intensity categorization
Gollan et al. (36)	To investigate how depressed patients interpret facial affect of differing intensity	Case-control Clinical sample	MDD = 44, HC = 44 [29.5 ± 9.8; 33M:55F]	PFA	DSM-IV	No significant main effect for group (MDD vs. HC) on facial affect recognition accuracy
Joorman and Gotlib (37)	To examine depression-specific biases in identification of affective facial expressions	Case-control Outpatient sample	MDD = 23, SP = 27, HC = 26 [31.9 ± 9.4; 21M:51F]	FEEST	DSM-IV	MDD, SP, and HC performed equally on facial affect recognition
Matthews et al. (38)	To examine amygdala-cingulate functional coupling in depression during an emotional face matching task	Case-control Community sample	MDD = 15, HC = 16 [24.4 ± 5.3; 9M:22F]	PFA	DSM-IV	No difference between MDD and HC for accuracy or reaction time on face matching task
Seidel et al. (39)	To measure automatic behaviors toward affective facial expressions in depression	Case-control Inpatient sample	MDD = 24, HC = 24 [42.4; 24M:24F]	VERT-K	DSM-IV	MDD and HC performed equally well on facial affect recognition
Suslow et al. (40)	To examine spatial detection of facial emotion in depressed inpatients undergoing psychotherapy	Prospective longitudinal case-control Inpatient sample	MDD = 11, MDD/AD = 11, HC = 22 [32.1 ± 8.3; 14M:30F]	FITCT	DSM-IV	Both MDD groups performed equally to HC in spatial detection of facial affect, at two time points
						Performance did not significantly improve in either group over the 7 weeks
Suslow et al. (41)	To assess awareness of masked facial expressions and automatic amygdala responses in depression	Case-control Inpatient sample	MDD = 30, HC = 26 [37.5 ± 12.4; 29M:27F]	PFA	DSM-IV	MDE and HC did not differ in performance when rating the valence of the masked facial expressions
Wilbertz et al. (22)	To explore preoperational features of ToM in depression	Case-control Clinical sample	MDD = 16, HC = 16 [43.7 ± 11.2; 16M:16F]	MASC	DSM-IV	MDD and HC performed equally on MASC multiple choice (*t*_30_ = 0.01, *P* = 0.924) and open answers (*t*_30_ = 0.03, *P* = 0.980)

*AD, anxiety disorder; BCPS, Brüne’s cartoon picture story test; BDI-II, Beck depression inventory-II; DSM-IV, diagnostic and statistical manual of mental disorders-IV; FEEST, facial expressions of emotion: stimuli and tests; FEPT, facial emotion and perception test; FERT, facial expression recognition task; FITCT, face-in-the-crowd task; FOQ, first order question; FPT, faux pas task; GP, general practice; HC, healthy controls; HRSD, Hamilton rating scale for depression; ICD-10, international classification of diseases, 10th revision; LEAS, levels of emotional awareness scale; MASC, movie for the assessment of social cognition; MA ± SD, participants’ mean age and standard deviation; MDD, major depressive disorder; MDE, major depressive episode; M:F, ratio of male to female participants; Mini-SEA, mini-social cognition and emotional assessment; N, number of participants; npMDD, non-psychotic major depressive disorder; PFA, Ekman and Friesen’s pictures of facial affect; pMDD, psychotic major depressive disorder; rMDD, remitted major depressive disorder; prMDD, major depressive disorder with psychosis risk; RMET-R, reading the mind in the eyes task, revised; sFPT, shortened faux pas task; SOQ, second-order question; SP, social phobia; ToM, theory of mind; WE.EL, Werden and Elikann test; VERT-K, Vienna emotion recognition tasks; VHI, virtual human interface; V-SIR, Versailles – situational intention reading*.

#### Facial affect recognition

The way in which depressed patients respond to emotional facial expressions can be broadly categorized according to valence of the emotion.

For neutral expressions, depressed patients appear to assign more negative interpretations than healthy controls (23, 30, 35), although this was not a consistent result. In some studies, depressed individuals were found to be significantly more likely to interpret a neutral stimulus as being sad (23, 35, 36, 42). In two small case-control studies involving 66 and 36 participants, respectively, Gollan et al. (35) and Leppänen et al. (30) found depressed participants identified more neutral expressions as sad than their non-depressed counterparts.

A similar bias is evident with respect to negatively valenced expressions (31), with some authors observing particular deficits for the expressions of sadness (36, 42) and fear (43). In one large case-controlled study of 230 participants, Anderson et al. (23) noted a significant bias toward the identification of negative expressions, namely anger and sadness, by those with a current or past history of depression in comparison to control participants. A case study by Csukly et al. (24) found a significant reduction in the accuracy of both sad and neutral facial recognition by the depressed patient group.

Studies examining the interpretation of positively valenced expressions – such as happiness – in a depressed population show an even greater discrepancy between results. There is some evidence to suggest that patients with major depressive disorder are less likely than healthy controls to correctly identify positive emotions (25, 31), however, this is far from a consistent finding. In a case-control study of 130 inpatients, Csukly et al. (25) found a significant negative association between recognition of happy expressions and presence of a maladaptive schema, however no other associations of significance were found with any of the other studied expressions including sadness. A similar study involving 56 inpatients by Surguladze et al. (31) also demonstrated a link between severity of depression and difficulty recognizing happy expressions, but in contrast observed an even greater impairment in recognition of negative expressions.

Another important factor in social cognitive performance is the ability to detect subtle expressions of facial emotion. Several smaller case-control studies (44–66 participants) incorporated variations of intensity into the assessment of facial affect recognition. Patients with major depressive disorder were identified as more likely to misattribute the expression as being of a higher intensity (35). Furthermore, depressed patients were found to require a greater intensity of emotion than their non-depressed counterparts to identify happy expressions (37, 44) and less intensity to identify sad expressions (36, 37, 42). Although the different research groups used a variety of different assessment tools, the degree of expression intensity may represent an important trend emerging within the body of literature.

Many studies incorporated assessment of reaction time, in addition to accuracy, for facial affect recognition. Several case-control studies showed individuals with major depressive disorder to have slower recognition of facial expressions than healthy controls (30, 31) and depressed patients with co-morbid anxiety to be slower in recognizing positive faces than both controls and non-anxious depressed (40). In a moderately sized case-control study, depressed patients were found to be significantly poorer at recognizing facial expressions when presented with an affective stimulus for 100 milliseconds, but equal to healthy controls when the time of stimulus was increased to 2,000 milliseconds (31). In contrast, reaction times were increased in some studies when depressed patients were confronted with sad (35) or neutral (30, 40) expressions. However, these represented a minority of results with the larger portion of investigators reporting no difference in reaction time (23, 28, 38, 41), throwing doubt over any potential association.

There were little data available investigating discrepancies in the way depressed and non-depressed subjects react to emotional stimuli. One group of researchers observed that depressed individuals demonstrate more avoidant behavior on an implicit joystick task when confronted by both negative and positive emotional stimuli, even when this information was correctly interpreted by the participant (39, 45). However, these studies both involved small numbers of participants (30 and 48, respectively) and only looked at instinctive reaction to facial expression without investigation of functional implications.

#### Affective theory of mind

There was greater consistency in the literature regarding affective theory of mind in major depressive disorder. Almost all studies employed the *Reading the Mind in the Eyes* assessment task (46), which required participants to interpret the affective mental state portrayed in various cropped images of eyes. The clear majority of studies found that depressed patients were impaired in this skill compared to controls (8, 21, 27, 29, 32). However, a separate study found depressed patients to be in fact more accurate than matched controls in identifying a negative emotional state, without any difference for neutral and positive states (2). This may indicate a bias toward negative emotions similar to the phenomenon observed with interpretation of facial expressions.

The only two studies that did not reveal a difference between groups on theory of mind performance indicated instead that the depressed group showed less perceptual reasoning than controls for the affective rather than cognitive components of theory of mind (2, 22). This preference against affective theory of mind was also demonstrated by Lee et al. (29) in a case-control study of 101 patients. Such findings may suggest that theory of mind deficits in major depressive disorder may be primarily on an emotional level.

A number of authors considered the effect of certain clinical features of depression on affective theory of mind performance, linking suicidal behaviors (47), excessive rumination (48), and anxiety (29) to impaired performance compared to patients who did not feature these symptoms. Additionally, patients with psychotic features of depression were significantly worse than depressed patients without these features (8, 21), which is in keeping with previous literature on psychotic disorders (1). However, risk of psychosis itself was not significant (32).

#### Cognitive theory of mind

A number of researchers also chose to use tools based on cognitive theory of mind ability in their assessment of possible social cognition impairments in depressed patients, with some evidence to suggest that patients with major depressive disorder have difficulties with these skills in comparison to healthy controls. In a case-control study involving 60 patients, Zobel et al. (33) found that depressed patients appear to be impaired on the key cognitive theory of mind tasks of interpreting both first- and second-order questions relating to social interactions. Further case-control studies by Cao et al. (21) and Wang et al. (8) also identified deficits amongst depressed patient populations in identifying social faux pas. However, as both these researchers chose to include psychotic depressed patients as a third subgroup as well as non-psychotic depressed patients and healthy controls, interpretation of these findings was difficult. Lastly, patients with major depressive disorder also showed poorer emotional awareness of what others were thinking compared to controls, but had good insight into their own emotional state (26). This was also manifest as a difficulty in showing empathy toward the feelings of others (22).

In contrast, there were also a number of studies that found little difference in cognitive theory of mind performance between the depressed and non-depressed. Wolkenstein et al. (2) found depressed patients to have difficulty integrating contextual information about other people or sequences of events, but was unable to identify any other significant theory of mind deficit. Three other small case-control studies found depressed and healthy participants to have similar outcomes in their assessments (7, 20, 22).

### Social cognition in acute and remitted depressive states

Some authors also examined whether acutely depressed patients performed differently on social cognitive tasks compared to those with remitted major depressive disorder. A very small sample base was retrieved, with the three relevant studies being detailed in Table 2. Anderson et al. (23) found that patients in remission were more likely to identify anger compared to controls, while Bhagwagar et al. (43) obtained a similar result for fear only. This most likely indicates priming for negative emotions, consistent with the previously reported finding that depressed patients required less intensity to identify negative expressions (23, 36, 42). LeMoult et al. (44) conducted a case-control study of 95 women, which indicated that patients with remitted major depressive disorder made fewer errors than controls on facial affect recognition overall, but required significantly greater emotional intensity to identify happy expressions than controls (p < 0.01).

**Table 2:** **Case-control studies investigating social cognitive performance in remitted major depressive disorder**.

Reference	Aim	Method	*N* [MA ± SD; M:F]	Social cognition task	Mood diagnosis	Results
Anderson et al. (23)	To compare accuracy, discrimination, and bias in face recognition in current and remitted depression	Case-control GP sample	MDE = 30, rMDD = 99, HC = 101 [33.1 ± 10.5; 71M:159F]	FERT	DSM-IV	rMDD more accurate on facial affect recognition for anger compared to HC (*P* < 0.05) and anger, fear and sadness compared to MDE (*P* < 0.01)
Bhagwagar et al. (43)	To assess facial affect recognition in depression and the affects of citalopram on performance	Randomized, placebo-controlled, double-blind, between-group Clinical sample	rMDD = 20, HC = 20 [37.3 ± 3.7; 0M:80F]	FERT	DSM-IV	rMDD showed a selectively greater recognition of fear relative to HC (*F*_1,18_ = 6.7, *P* = 0.02), but not for other expressions
LeMoult et al. (44)	To investigate the identification of affective facial expressions in remitted depression	Case-control Community sample	rMDD = 39, HC = 56 [43.5 ± 5.6; 0M:95F]	FEEST	DSM-IV	rMDD performed better than HC on facial affect recognition (*F*_1,93_ = 4.96, *P* < 0.05) and required significantly greater emotional intensity to identify happy expressions than HC (*t*_93_ = 3.34, *P* < 0.01)

*DSM-IV, diagnostic and statistical manual of mental disorders-IV; FEEST, facial expressions of emotion: stimuli and tests; FERT, facial expression recognition task; HC, healthy controls; ICD-10, international classification of diseases, 10th Revision; MA ± SD, participants’ mean age and standard deviation; MDE, major depressive episode; M:F, ratio of male to female participants; N, number of participants; rMDD, remitted major depressive disorder*.

The relationship between acute and remitted major depressive disorder can also be considered in terms of treatment. A number of studies have considered the role of intervention, with one randomized controlled trial finding that acute administration of citalopram improved facial affect recognition accuracy of fear compared to placebo (43). Emotional awareness of others also improved following a course of inpatient psychoanalytic-interactional group therapy (26), indicating the potential utility of non-pharmacological approaches. This evidence suggests that the alterations in social cognitive ability that occur with depression are reversible if the depressive state is treated. This is supported by the observation of another study that impaired performance in the depressed group was confined to untreated patients and that depressed subjects taking anti-depressant medication performed equally to controls (23). Clinical improvement may also be a function of dosage, as one study observed that higher doses of anti-depressants produced a tendency to rate fewer expressions as sad (31).

Two longitudinal studies of serially assessed patients with no therapeutic intervention have found that response accuracy did not change over time (40, 42). Indeed, it was shown that labeling performance remains constant, even when symptom severity was seen to decrease significantly in the patient group over a 6-month period (42). This stable impairment in facial affect recognition may indicate that depressed patients are vulnerable to social problems. Additionally, these investigations suggest that some of the variability seen in the results between groups may be due to depressed population samples being on pharmacotherapy whilst being tested. Longitudinal and interventional studies with a greater follow-up time would be useful however, as all these studies were quite short.

### Effect of severity of depression on social cognitive performance

Social cognitive performance in major depressive disorder is linked to the severity of depressive symptoms, as described in Table 3. In particular, there is a significant negative association between social cognitive performance and a higher score on a validated depression severity scale (26, 29). A study that separated the depressed cohort into severe and mild/moderate groups found those in the severe category performed statistically poorer than controls, while the mild/moderate group were trend worse (29). Others have found an isolated association between increasing severity and a deficit responding to happiness (25, 31) or an advantage for recognizing sadness (30, 36).

**Table 3:** **Association between severity of depressive symptoms and social cognitive performance**.

Reference	Aim	Method	*N* [MA ± SD; M:F]	Social cognition Task	Diagnosis and severity	Results
Cao et al. (21)	To investigate social cognitive performance in esophageal cancer patients with depression	Case-control Esophageal cancer patient sample	pMDD = 32, npMDD = 33, HC = 62 [33.1 ± 10.5; 71M:159F]	RMET-R, FPT	BDI-II, BPRS	In MDD, psychotic symptoms were negatively correlated with performance on both RMET-R (*r* = −0.35, *P* < 0.01) and FPT (*r* = −0.51, *P* < 0.01)
Csukly et al. (25)	To identify associations between depressive severity, maladaptive schemas, and facial affect	Case-control Inpatient sample	MDD = 107, HC = 23 [41.1 ± 11.3; 16M:90F]	VHI	DSM-IV, GSI, BDI	One SD increase in GSI was associated with decreased overall percentage recognition (*B* = −0.03). Happiness recognition was negatively associated with both BDI (χ^2^ = 8.6, *P* = 0.004) and SCL90-D (χ^2^ = 7.9, *P* = 0.03)
Derntl et al. (45)	To investigate the neural correlates of approach and withdrawal to affective faces in depressed patients	Case-control Inpatient sample	MDD = 15, HC = 30 [33.5 ± 10.3; 12M:18F]	VERT-K	DSM-IV, BDI, HRSD	MDD with higher HRSD scores showed less approach to happy faces (*r* = −0.602, *P* = 0.017) and more avoidance to angry faces (*r* = 0.725, *P* = 0.002)
Donges et al. (26)	To examine emotional awareness in depressed inpatients following a psychotherapy program	Prospective longitudinal case-control Inpatient sample	MDD = 22, HC = 22 [32.1 ± 8.6; 14M:30F]	LEAS	DSM-IV, BDI, ATQ	At baseline, degree of emotional awareness of others correlated with BDI (*r* = −0.29, *P* < 0.05) and ATQ (*r* = −0.27, *P* < 0.05)
						At 7-week follow-up, there was no correlation between performance and severity scores
Gollan et al. (36)	To investigate how depressed patients interpret facial affect of differing intensity	Case-control Clinical sample	MDD = 44, HC = 44 [29.5 ± 9.8; 33M:55F]	PFA	DSM-IV, HRSD	Depressive severity on HRSD was negatively correlated with recognition accuracy for sad faces (*r*_44_ = 0.29, *P* = 0.05), but not other emotions
Lee et al. (29)	To examine depressed patients’ abilities to identify mental states from affective eye expressions	Case-control Outpatient sample	mMDD = 15, sMDD = 37, HC = 30 [42.7 ± 14.1; 0M:82F]	RMET-R	DSM-IV	sMDD significantly less accurate than HC on affective ToM task (*t*_65_ = 2.24, *P* = 0.03); while mMDD were less accurate at a trend level (*t*_43_ = 1.66, *P* = 0.10)
Leppänen et al. (30)	To determine if depression biases the recognition of emotionally neutral faces	Case-control Inpatient sample	MDD = 18, HC = 18 [44.9 ± 9.9; 14M:22F]	PFA	ICD-10, BDI	Increased BDI scores were correlated with the proportion of incorrect ratings of neutral faces as sad (*r*_36_ = 0.60, *P* < 0.001)
Milders et al. (42)	To investigate the stability of emotion recognition impairments over 3 months in depressed patients	Prospective longitudinal case-control Clinical sample	MDD = 19, HC = 25 [46.9 ± 11.5; 15M:29F]	PFA	ICD-10, BDI-II, HRDS	Sadness recognition was not associated with symptom severity on HRSD or BDI-II, or decreasing severity over time (*P*s < 0.10)
Raes et al. (48)	To determine if rumination is associated with negative interpretation of facial affect in depression	Cross-sectional Clinical sample	MDD = 26 [39.6 ± 10.9; 9M:17F]	PFEQ	DSM-IV, BDI	Rumination score correlated with perception of negative facial affect (*r* = 0.51, *P* < 0.001), but not positive
						No correlation with BDI score for either
Surguladze et al. (31)	To investigate the accuracy and response bias of depressed people to affective facial expressions	Case-control Clinical sample	MDD = 27, HC = 29 [45.0 ± 11.6; 24M:32F]	FEEST	DSM-IV, HRSD, BDI	HRSD correlated with identification of sadness at 100 ms (ρ = −0.44, *P* < 0.05) and 2,000 ms (ρ = −0.48, *P* < 0.05), while BDI correlated with happiness at 100 ms (ρ = −0.49, *P* < 0.05) and sadness at 2,000 ms (ρ = −0.52, *P* < 0.01)
Szanto et al. (47)	To assess the relationship between affective ToM, problem solving, social functioning and suicide in late-life depression	Case-control Clinical sample	sbMDD = 24, nsbMDD = 38, HC = 28 [69.3 ± 7.6; 39M:51F]	RMET-R	DSM-IV, HRSD, BSSI	sbMDD impaired on affective ToM task compared to HC (*F*_2,87_ = 4.25, *P* = 0.017), while nsbMDD were no different to HC
Wang et al. (8)	To determine if psychotic features in depression affect social cognitive performance	Case-control Inpatient sample	pMDD = 23, npMDD = 33, HC = 53 [26.8 ± 4.4; 47M:62F]	RMET-R, FPT	ICD-10	In MDD, psychotic symptoms were negatively correlated with both RMET-R (*r* = −0.357, *P* = 0.007) and FPT (*r* = −0.475, *P* < 0.000)
Wolkenstein et al. (2)	To investigate difference in social cognitive performance between depressed patients and controls	Case-control Outpatient sample	MDD = 24, HC = 20 [36.4 ± 10.8; 19M:25F]	RMET-R, MASC	DSM-IV, QIDS	QIDS correlated with selecting “no ToM’ responses on MASC (*r* = −0.54, *P* < 0.01), but RMET-R was not associated with QIDS

*ATQ, automatic thoughts questionnaire; BDI, Beck depression inventory; BDI-II, Beck depression inventory-II; BPRS, brief psychiatric rating scale; BSSI, Beck scale for suicidal ideation; DSM-IV, diagnostic and statistical manual of mental disorders-IV; GSI, global severity index; FEEST, facial expressions of emotion: stimuli and tests; FPT, faux pas task; HC, healthy controls; HRSD, Hamilton rating scale for depression; ICD-10, international classification of diseases, 10th revision; LEAS, levels of emotional awareness scale; MA ± SD, participants’ mean age and standard deviation; MDD, major depressive disorder; M:F, ratio of male to female participants; MASC, movie for the assessment of social cognition; mMDD, mild/moderate major depressive disorder; N, number of participants; npMDD, non-psychotic major depressive disorder; nsbMDD, major depressive disorder with no suicidal behavior; PFA, Ekman and Friesen’s pictures of facial affect; PFEQ, perception of facial expressions questionnaire; pMDD, psychotic major depressive disorder; QIDS, quick inventory of depressive symptoms; RMET-R, reading the mind in the eyes task, revised; sbMDD, major depressive disorder with suicidal behavior; SCL90-D, symptom checklist 90 depression subscale; SD, standard deviation; sMDD, severe major depressive disorder; ToM, theory of mind; VERT-K, Vienna emotion recognition tasks; VHI, virtual human interface*.

These findings remain consistent with previous evidence suggesting a negative interpretative bias in emotional recognition. Theory of mind ability is also affected, as one study showed that increasing depressive severity was associated with responses that showed poor understanding of mental states (2).

In addition to severity of symptoms overall, the presence of certain severe symptoms also has an impact. Suicidal behavior and psychotic features are features that indicate a severe depressive illness and, as reported previously, both strongly correlate with the impaired interpretation of social stimuli (8, 21, 47). Therefore, the phenotype of the illness is important in addition to the burden of symptoms.

The link observed between social cognitive performance and severity of major depressive disorder could also help explain the discrepancies observed in the group difference results, as studies that have a high proportion of milder depression cases may not achieve significant results. Nevertheless, there were also some studies that found no association with symptom severity (42), while another showed a negative association between severity and sadness accuracy (31).

## Discussion

This review of the literature demonstrates a number of emerging trends in the way that individuals with major depressive disorder process and interpret socially salient information. Importantly, a negative bias may exist in the way that depressed subjects recognize emotion, which manifests as performance differences according to valence of emotion. Additionally, the review indicates that social cognitive performance may be related to severity of the depressive illness, with more pronounced deficits being associated with increased scores on depressive symptom scales. Some authors have suggested that depressed subjects may require greater intensity of emotion and a longer reaction time when interpreting emotional stimuli compared to controls.

The literature, however, remains equivocal regarding many aspects of the relationship of social cognition and major depressive disorder, with a paucity of evidence in the domains of prosody and body language as well as limited data investigating patients in clinical remission. A noteworthy number of studies do not support these trends with some even finding no observed group differences between the depressed and controls in overall performance on tests of social cognitive function. This would suggest that any deficits in depression are subtler than in other disorders and may not be identified by broad measures of functioning.

One explanation for this variation in findings could be the predisposition of depressed patients to negatively interpret social stimuli. This bias would appear to improve performance for negatively valenced stimuli whilst simultaneously impairing performance for positively or neutrally valenced stimuli. The net effect of this bias could reduce statistical significance for overall performance on a task. Thus, the true differences in facial affect recognition may be masked if valence is not taken into account.

This observed negative interpretive bias on social cognitive tasks is mood-congruent and consistent with the cognitive paradigm of depression, as proposed by Beck (49, 50). This conceptualization of depression proposes that the thoughts, interpretations, and attitudes of an individual play an important role in the pathogenesis of the disorder. As major depressive disorder severely affects emotional states, it would fit that the deficits in social cognition would predominantly occur in the emotional domain, rather than in the cognitive skills of interpreting beliefs and intentions. The trends emergent in this review would support this, as the evidence is far more conclusive from tests assessing the emotional domain of social cognition, through both facial affect recognition and affective theory of mind. However some uncertainty remains, as there is little data available in cognitive theory of mind on which to base this assertion.

Few authors have addressed the important conceptual aspect of general cognition. Several cognitive domains, such as attention, memory, and executive functioning, have previously been found to be impaired in major depressive disorder (51, 52), implying that cognitive deficits may play a role in the pathophysiology of not only depression but also the associated impairments in general functioning or quality of life. General cognitive skills are crucial for the performance in tasks of social cognition tasks and their role is an important, unquantified factor in the studies covered by this review.

Indeed, difficulties with social interaction observed in patients with major depressive disorder may, at least in part, be due to an impaired ability to interpret emotional stimuli and mental states. In fact, social cognitive performance in depression may impact on the development of the disorder through impairing social functioning. Inaccurate assessments of feedback from interpersonal interactions exacerbate maladaptive thought processes, ultimately affecting mood. This could then diminish motivation to engage in social interaction, which would reduce reward from socializing and further intensify isolation. Awareness of social cognitive ability could be a beneficial component in cognitive-based psychotherapies.

### Methodological limitations

The discrepancies in results between some studies may be due to the variety of different tools employed to measure social cognition. Multiple measures exist for each component of social cognition and the tools are often tailored to the specific hypotheses of the investigation. This prevents accurate comparison between results, as well as limiting the reproducibility. Additionally, the majority of studies on social cognition use a unimodal design that exclusively focuses on one domain of social cognition, most commonly emotion recognition. There would be benefit in employing a study design that tests multiple domains in the same population. Some components of social cognition, such as prosody or body language, would also benefit from further directed research in major depressive disorder. It is difficult to make confident conclusions from only a small collection of studies.

Even the term “social cognition” is not universally used throughout the literature, with some authors preferring “social perception” (1, 4). This also creates a challenge when searching the literature, as different databases categorize the literature under different headings – Medline uses “social perception,” Embase uses “social cognition,” and PsychINFO uses both. The search is further complicated by the large number of individual social cognitive domains, meaning that some relevant articles may have been overlooked if not linked under the headings of social cognition or social perception. This is evident through the relatively large number of additional studies indentified for this review through the reference lists of included papers. A more targeted search with specific keywords identifying “prosody” and “theory of mind” may have also yielded greater results.

### Future directions

A clear need exists for further research, particularly in domains such as prosody and body language interpretation, which have not yet been well-described in a major depressive disorder population. In general, larger longitudinal studies of major depressive disorder would be helpful in furthering understanding of the relationship between acute and remitted depression, as well as response to treatment. Agreement on or standardization of both terminology and testing in the area of social cognition would be a helpful advent and allow for greater reproducibility and ease for comparison of results.

Understanding the relationship between major depressive disorder and social cognitive performance is important in characterizing both the etiology and phenotypology of major depressive disorder. In terms of potential implications for therapy, the results of two of the interventional studies included in this review indicated that treatment with anti-depressants, in particular citalopram, had a normalizing effect for a number of the observed social cognition defects of facial affect recognition. These findings suggest that the changes to social cognitive skills in depression are reversible. Despite being amenable to pharmacotherapy, it has not yet been established whether the observed findings impact on daily functioning and quality of life independent of that from depression. It is therefore important to quantify the functional burden, if any, of impaired social cognitive performance in major depressive disorder to determine its specific clinical relevance. Further research in this area would also be pertinent.

## Conclusion

Patients with major depressive disorder appear to interpret emotional stimuli differently to healthy controls, although this is not a consistent finding throughout the recent literature. The difficulties with social interaction and functioning observed in depressed patients may, at least in part, be due to an altered ability to correctly interpret emotions or mental states. It appears that depressed patients may struggle more with subtle or nuanced expressions of emotion, as well as demonstrating a mood-congruent bias for interpreting stimuli more negatively. These features are linked with severity of the illness and may persist even in the remitted state. However, it appears that some impairments are at least partially reversible using anti-depressants or psychotherapy. Further research is required in this area to better understand the functional impact of these findings and the role of targeted therapy in improving how depressed individuals interact socially.

## Conflict of Interest Statement

The authors declare that the research was conducted in the absence of any commercial or financial relationships that could be construed as a potential conflict of interest.
